# Factors influencing phagocytosis of malaria parasites: the story so far

**DOI:** 10.1186/s12936-021-03849-1

**Published:** 2021-07-16

**Authors:** Caroline Lin Lin Chua, Ida May Jen Ng, Bryan Ju Min Yap, Andrew Teo

**Affiliations:** 1 School of Biosciences, Taylor’s University, Subang Jaya, Selangor Malaysia; 2 Lee Kong Chian School of Medicine, Nanyang Technological University, Singapore, Singapore; 3 Department of Medicine, The Doherty Institute, University of Melbourne, Victoria, Australia

**Keywords:** Phagocytosis, Opsonic, Non-opsonic, Monocytes, Macrophages, Neutrophils, Antibody

## Abstract

There are seven known species of *Plasmodium* spp. that can infect humans. The human host can mount a complex network of immunological responses to fight infection and one of these immune functions is phagocytosis. Effective and timely phagocytosis of parasites, accompanied by the activation of a regulated inflammatory response, is beneficial for parasite clearance. Functional studies have identified specific opsonins, particularly antibodies and distinct phagocyte sub-populations that are associated with clinical protection against malaria. In addition, cellular and molecular studies have enhanced the understanding of the immunological pathways and outcomes following phagocytosis of malaria parasites. In this review, an integrated view of the factors that can affect phagocytosis of infected erythrocytes and parasite components, the immunological consequences and their association with clinical protection against *Plasmodium* spp. infection is provided. Several red blood cell disorders and co-infections, and drugs that can influence phagocytic capability during malaria are also discussed. It is hoped that an enhanced understanding of this immunological process can benefit the design of new therapeutics and vaccines to combat this infectious disease.

## Background

Phagocytosis is a process that involves cellular uptake of particles to mediate the clearance of apoptotic cells and invading micro-organisms. Professional phagocytes such as monocytes, macrophages and neutrophils can be activated to efficiently execute this immune function. Based on the mechanisms of phagocytic target recognition, phagocytosis can be categorized into either opsonic or non-opsonic (as reviewed by Uribe-Querol and Rosales [1]). Opsonic phagocytosis involves the coating of antigens with host proteins (opsonins) such as C3b or C4b complement fragments, antibodies and collectins, followed by binding of these complexes to phagocytic receptors on immune cells to promote internalization. In non-opsonic phagocytosis, phagocytic targets bind directly to surface receptors such as CD36, toll-like receptors (TLRs) and complement receptor 3 to induce uptake. These receptors recognize pathogen-associated molecular patterns of the microbes, which are molecules that are exclusively expressed by the microbes but not by host cells [2].

Using in vitro models, it was demonstrated that both opsonic and non-opsonic phagocytosis of *Plasmodium* spp. can be carried out by phagocytes, particularly monocytes and macrophages [3–5]. However, much less understood are the mechanisms that can promote in vivo activation of phagocytes leading to phagocytosis and the downstream phagocytic pathways in patients with malaria. While the phagocytosis of *Plasmodium*-infected erythrocytes (IEs) is commonly observed in vitro, evidence of phagocytosis in vivo is mainly characterized by the presence of haemozoin within phagocytes [6–8]. Haemozoin is the end product of haemoglobin digestion by parasites and it is well known for its immunomodulatory activities, including the ability to inhibit further phagocytosis of IEs by macrophages that have previously ingested haemozoin [9]. In contrast, little is known about the mechanisms involved in the uptake of different parasite stages such as sporozoites, merozoites, schizonts and gametocytes, and the downstream molecular consequences. In addition, there are many populations and sub-populations of phagocytes, but their relative importance in the phagocytosis of parasites during malaria is not well studied.

In this review, current knowledge on factors that may enhance or inhibit phagocytosis of *Plasmodium* spp. and their association with clinical outcomes are reviewed. Additionally, the distinct signalling pathways that are activated following phagocytosis, which may in turn influence the inflammatory and clinical outcomes, are also discussed [10–12]. *Plasmodium falciparum* is the most deadly and prevalent species in Africa, where malaria burden is also the highest, hence the majority of discussions are focussed on this species, unless specified otherwise. To conclude, several key pathways that can modulate phagocytic function of phagocytes and some important questions for future research are proposed. A better understanding of these molecular and cellular interactions can benefit the design of vaccines and adjunct interventions that enhance anti-parasitic responses in malaria.

## *Plasmodium* spp.: the encounter with phagocytes in infected human host

Throughout the life cycle of *Plasmodium* spp., the parasites may encounter different populations of phagocytes in the infected human host. In the initial stage of infection, parasites are injected into the host in the form of sporozoites by *Anopheles* mosquitoes, and the sporozoites then travel to the liver. After replication in the liver cells, they develop into merozoites, which are released into the blood circulation to invade host erythrocytes. Subsequently, the parasites may develop into either gametocytes or the immature ring-stage trophozoites, followed by mature trophozoites, schizonts and merozoites. Sporozoites within the liver encounter liver macrophages, known as Kupffer cells, whereas the blood-stages of parasites may encounter circulating host monocytes and neutrophils. Mature trophozoites within IEs can be circulated into organs, such as the brain, spleen, placenta, and lung, and sequester within these organs as part of an immune evasion strategy [13]. At this stage, they will encounter tissue macrophages and other circulating phagocytes that have been recruited in response to the local infection [14].

While many studies have shown that professional phagocytes can phagocytize the asexual stages of the parasites [3, 5, 15], there are contrasting data on the ability of phagocytes to phagocytize gametocytes, the sexual stage parasites. Gametocytes undergo five stages of development from stage I to stage V; early stage gametocytes can be found in the bone marrow, while late stage V gametocytes can be found in peripheral blood [16]. In the absence of opsonins, human monocytes and macrophages were shown to phagocytize the early stage I and II gametocytes, while mouse bone marrow-derived macrophages were reported to phagocytize early and mature stages of gametocytes; both involved in vitro studies [17, 18]. In contrast, THP-1 macrophages did not phagocytize live, unopsonized and intra-erythrocytic mature stage V gametocytes [19]. Gametocytes were previously detected in the extracellular environment within the bone marrow, hence they were proposed to be resistant to phagocytosis in vivo [20]. The use of different phagocyte models that may have different expression levels of phagocytic receptors such as CD36 can potentially account for these discordant findings; nonetheless, more studies are required to ascertain this.

In this review, the focus will be on the phagocytosis of asexual stage of the parasites, given that a majority of the data on phagocytosis are based on parasites at this stage. There are many factors that may determine if malaria parasites are phagocytized, including the presence and types of opsonins generated in *Plasmodium*-infected hosts, the phenotypes and activation states of host phagocytes, the expression levels of phagocytic cell receptors, and the mechanisms of phagocytosis, whether opsonic or non-opsonic [5, 21, 22]. These are discussed in more detail in subsequent sections.

## Factors that influence phagocytosis during *Plasmodium* spp. infection

### Opsonizing antibodies: isotypes and specificities

Antibodies targeting different stages of the parasite’s life cycle have various functions during infection, which include neutralization, antibody-dependent cell inhibition and opsonization. However, to act as an opsonin and promote effective phagocytosis, the antibodies must be of particular isotypes, subclasses and specificities. Using in vitro assays, non-cytophilic antibodies such as IgG4 and IgM were reported to decrease opsonic phagocytosis of sporozoite circumsporozoite protein (CSP) and IEs, respectively [23, 24]. On the other hand, protective immunity in malaria was mainly associated with IgG1 and IgG3 subclasses (reviewed in [9]). These two antibodies are cytophilic in nature, where they can bind with strong avidity and affinity to antigens via their Fab region, and to immune cells expressing Fc receptors via their Fc region.

The opsonizing function of antibodies and the levels of opsonizing antibodies in malaria patients were mainly studied using in vitro phagocytosis assays, which often involve the use of infected-patient sera, cultured parasites and phagocytes. Undifferentiated and differentiated THP-1 monocytic cells are commonly used as the phagocytes in these assays due to convenience in maintaining cell culture, although there are limitations, such as differential expression of phagocytic receptors between these cells and actual circulating phagocytes, which can result in different phagocytic capacity [25]. Antibodies that can specifically induce the phagocytosis of parasites in vitro, referred to as ‘opsonizing antibodies’ in this review, can be quantified from phagocytosis assays and their correlation with protection can be investigated.

Opsonizing antibodies targeting different stages in the asexual life cycle of the parasites have been associated with protection. For example, opsonizing antibodies that target merozoites were associated with protection against clinical malaria and high parasitaemia [4, 26]. In adult volunteers who received the RTS-S vaccine, high levels of opsonizing antibodies targeting CSP were induced and these were associated with protection against clinical malaria [27, 28]. Similarly, infected children with higher level of opsonizing antibodies against IEs were less likely to develop severe malaria [29], while high levels of opsonizing antibodies against IEs in pregnant women have been associated with protection from severe malaria and maternal anaemia, suggesting their importance in preventing disease progression [30]. Despite these evidences of protection conferred by opsonizing antibodies, it is noteworthy that these antibodies can also participate in other anti-parasitic functions, such as antibody-dependent cell cytotoxicity and antibody-dependent cellular inhibition [31, 32]. Hence, the protection associated with antibody levels may or may not be solely attributed to their ability to increase phagocytosis in vivo, and different antibody functional assays need to be carried out to obtain a better understanding of mechanisms mediating protection.

Previous studies have identified various antigenic targets of naturally acquired opsonizing antibodies produced during *P. falciparum* infection, which can be useful in vaccine design. Specific proteins on the merozoites such as merozoite surface proteins (MSP) 2 and 3, MSP-Duffy binding-like proteins 1 and 2, and glutamate-rich protein have been identified as targets of these opsonizing antibodies [33–35]. *Plasmodium falciparum* erythrocyte membrane protein 1 (PfEMP1) expressed on the surface of IEs is also a major target of opsonizing antibodies [36], where the antibodies have been shown to recognize DBL2, DBL3, and DBL5ε domains of VAR2CSA, which is a member of PfEMP1 protein family [37]. Together, these observations suggest that specific antibodies are required to recognize unique antigens on the parasites for opsonic phagocytosis to take place.

## Heterogeneity in host phagocyte subpopulations: who is the big eater?

### Monocytes: the circulating phagocytes

#### Monocyte sub-set

**Fig. 1:**
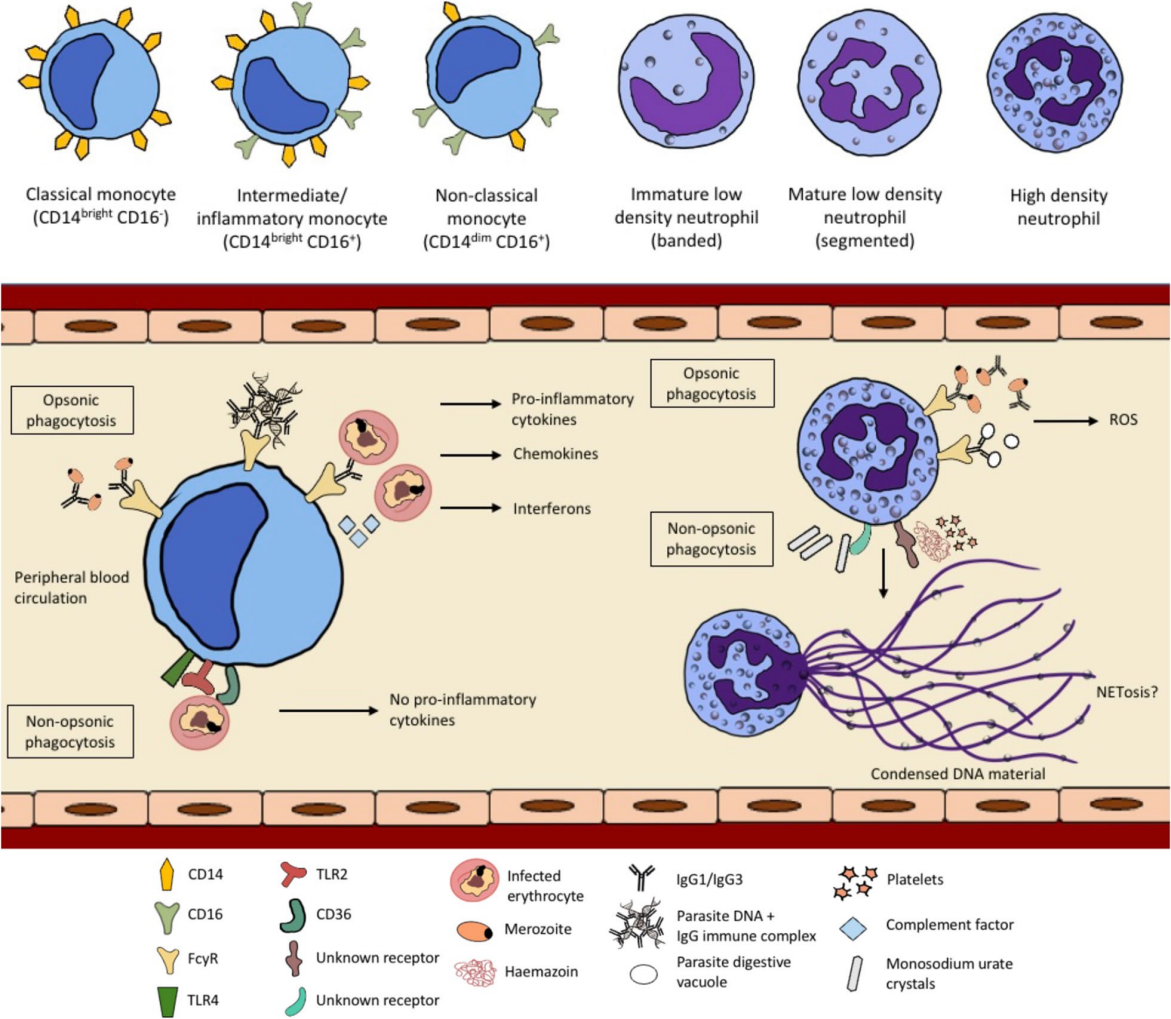
Phagocytosis of *Plasmodium falciparum* by monocytes and neutrophils. Monocytes and neutrophils can phagocytize *P. falciparum* or other parasite-related products via non-opsonic phagocytosis and/or opsonic phagocytosis. Antibody-mediated opsonic phagocytosis, which involves cytophilic antibodies IgG1 and IgG3, is likely to exert protective effect by promoting parasite clearance [12]. There are three main sub-sets of monocytes according to their CD14 and CD16 receptor expression; classical, intermediate and non-classical. The intermediate/inflammatory and non-classical monocyte populations can be expanded during an infection and they are more efficient in the uptake of infected erythrocytes (IEs) for both opsonic and non-opsonic phagocytosis. Phagocytosis of opsonized IEs by monocytes requires the participation of Fcγ receptors and complement factors, while phagocytosis of unopsonized IEs involves the participation of CD36, TLR2 and TLR4 [43]. Opsonic phagocytosis of IEs, merozoites and parasite DNA by monocytes leads to the production of pro-inflammatory mediators, while non-opsonic phagocytosis does not [5, 11, 45, 46]. There are also different sub-sets of neutrophils; low and high-density neutrophils, but it is unknown if they have distinct ability in the phagocytosis of *Plasmodium*. Opsonic phagocytosis of merozoites and parasite digestive vacuoles by neutrophils can lead to reactive oxygen species (ROS) production [85]. The outcomes following phagocytosis of monosodium urate crystals and haemozoin (which requires the presence of platelets) by neutrophils during malaria are not known, but it can potentially result in NETosis; this requires further investigation

Apart from availability of opsonins, phagocytosis of parasites is also dependent on the recruitment of host phagocyte populations, which are often heterogeneous and comprise different sub-populations with distinct phagocytic potentials. Human monocytes can be classified into three major sub-sets based on CD14 and CD16 surface marker expression (Fig. 1). In the circulation of a healthy adult, approximately 90% of total monocytes are classical monocytes (also described as CD14^bright^CD16^−^), while the remaining 10% are CD16^+^ monocytes. The latter sub-set can be further classified into either intermediate/inflammatory monocytes (CD14^bright^CD16^+^) or non-classical monocytes (CD14^dim^CD16^+^), and these can be expanded during infection and inflammation. Previous studies have reported the expansion of these monocyte sub-sets in patients with malaria [10, 38–40].

### Monocyte sub-sets and their phagocytic function

Comparing across different monocyte sub-sets, the intermediate/inflammatory monocytes were reported to be most efficient in the opsonic phagocytosis of IEs in a previous study [5] (Table 1). In the study, the authors used monocytes from malaria-naïve individuals and challenged them with antibody-opsonized IEs in vitro; they also reported that this phagocytosis pathway requires the participation of complement and CD16, which is also known as FcγRIII that binds efficiently to IgG1 and IgG3 antibodies [41]. Using blood samples from Kenyan children with acute malaria, their intermediate/inflammatory and non-classical monocytes showed significantly higher opsonic phagocytosis of IEs compared to classical monocytes [10]. Interestingly, the intermediate/inflammatory monocytes from *Plasmodium vivax*-infected patients also have the highest phagocytic potential when challenged with *P. vivax*-infected reticulocytes [42]; however, not much is known about monocyte sub-sets in other human *Plasmodium* spp. infections.

**Table 1:** Different sub-populations of phagocytes and their phagocytic ability in malaria

Phagocyte population	Surface marker expression	Origin	Phagocytic ability		References
			Parasite species	Phagocytic ability	
Monocytes					
Classical monocytes	CD14^bright^CD16^−^	Human	*P. falciparum* IEs	Intermediate/inflammatory > non-classical > classical	[5, 10]
Intermediate/inflammatory monocytes	CD14^bright^CD16^+^				
Non-classical monocytes	CD14^dim^CD16^+^				
Macrophages					
Splenic macrophages	CD11b^high^CD14^+^F4/80^+^TCRβ^+^	Mouse	*P. berghei* IEs	Higher phagocytic potential compared to TCRβ^−^ macrophages	[53]
	CD11b^+^Tim3^+^	Mouse	*P. berghei* IEs	Reduced phagocytic ability due to Tim3 expression	[55]
Neutrophils					
TCRβ+ neutrophils	CD11b^+^ Ly6G^+^TCRβ+	Mouse	*P. berghei* IEs	Higher phagocytic potential compared to TCRβ^−^ neutrophils	[79]
Low density neutrophils	CD66b^+^CD16^+^CD11b^+^CD15^+^	Human	*P. vivax*-infected reticulocytes	Comparable phagocytic potential to high density neutrophils from malaria patients, higher phagocytosis ability compared to high density neutrophils from healthy donors	[76]

For non-opsonic phagocytosis, intermediate/inflammatory and non-classical monocytes from *P. falciparum*-infected children were shown in vitro to be more efficient in the uptake of IEs compared to classical monocytes, with more prominent effect observed 6 weeks after receiving artemether–lumefantrine treatment [10]. Importantly, this activity was highest in the intermediate/inflammatory monocytes, which have higher expression of CD36, TLR 2 and TLR4 [10]. High levels of the aforementioned receptors potentially account for their higher phagocytic activities, given that TLR stimulation was previously reported to enhance CD36-mediated non-opsonic phagocytosis [43].

To date, only a few studies investigated the relationship between each monocyte sub-set, their phagocytosis potential and association with protection during *Plasmodium* spp*.* infection. Increased levels of the highly phagocytic intermediate/inflammatory monocytes did not appear to be associated with protection as they have been reported in children with severe anaemia, suggesting that increased phagocytosis may instead contribute to pathology by accelerating erythrocyte destruction [39]. In contrast, in a recent study involving Beninese young children, lower levels of non-classical monocytes were found in patients who died from malaria and these reduced levels were associated with increased disease severity, characterized by development of severe anaemia and cerebral malaria [44]. However, whether or not this protection from mortality and disease severity is attributed to the monocytes’ high phagocytosis ability and their inflammatory responses remains to be determined.

### Monocyte activation following phagocytosis

The uptake of *P. falciparum* and its associated proteins by monocytes via distinct phagocytosis pathways can lead to different consequences. Non-opsonic phagocytosis of IEs by human peripheral monocytes via CD36 receptor does not induce tumour necrosis factor (TNF) production [45]. In contrast, opsonic phagocytosis appears to be associated with the activation of innate immune responses. Stimulation of monocytes from malaria-naïve individuals with antibody-opsonized IEs and purified parasite DNA-containing immune complexes can activate signalling pathways that lead to the production of pro-inflammatory cytokines, TNF and IL-1β [5, 11, 46]. Using *P. vivax-*patient samples, the intermediate/inflammatory monocytes have been identified as the main producers of pro-inflammatory cytokines upon stimulation with immune complexes [11]. Besides being associated with specific antibodies, parasite DNA can also be bound to haemozoin or be packaged in extracellular vesicles during parasite ring stage, prior to being phagocytized by monocytes [47, 48]. These extracellular vesicles may contain both genomic DNA and small RNA, and upon release into host cell cytosol, they can activate ‘stimulator of interferon genes’ (STING) [48]. STING is a cytosolic DNA sensor and was shown to induce the production of type I interferons and chemokines by THP-1 monocytes stimulated with parasite extracellular vesicles [48].

### Macrophages: the tissue phagocytes

#### Macrophage sub-sets and their phagocytosis function during malaria

Macrophages exist in a continuum of activation states and they are commonly classified into M1 and M2 macrophages. M1 macrophages mainly secrete pro-inflammatory cytokines such as TNF, IL-1 and IL-6, while M2 macrophages can produce high levels of anti-inflammatory cytokines such as IL-10 and TGF-β [49]. There are also other alternative sub-populations such as CD169^+^, T cell receptor (TCR)^+^ and tumour-associated macrophages [50], but these are less well understood compared to the M1 and M2 paradigm. In lung tissues of patients who died from *P. falciparum* infection with pulmonary oedema, increased numbers of lung macrophages exhibiting M1 phenotype (CD68^+^ CD40^+^) have been reported [51], suggesting their role mediating lung pathology. In other cases of severe malaria, such as cerebral and placental malaria, high levels of pro-inflammatory cytokines and growth factors, such as TNF, interferon (IFN)-γ and granulocyte/macrophage colony stimulating factor (GM-CSF) could potentially polarize tissue macrophages to M1 phenotype; this remains to be proven in future studies.

Due to the inaccessibility to human macrophages in different organs, most of the understanding of parasite–macrophage interactions are based on animal models. In a transgenic BALB/c mouse model where mice were depleted of CD169 using diphtheria toxin and infected with *Plasmodium berghei*, CD169^+^ tissue-resident macrophages that are usually found in the spleen, lymph nodes and liver sinusoids were reported to be crucial in limiting parasite sequestration, which otherwise can lead to systemic inflammation and multiple organ damage [52]. In another study using C57BL/6 mice, it was reported that a novel population of CD11b^high^CD14^+^F4/80^+^ macrophages that express combinatorial TCRβ receptor can be expanded during *P. berghei* infection [53]. These cells are found primarily in the brain and spleen during *P*. *berghei* infection and are highly efficient in the phagocytosis of IEs, a function attributed to the expression of TCRβ receptor [53]. In humans, a small proportion of monocytes (approximately 5%) also express TCRαβ [54] but its role in phagocytosis of parasites has yet to be determined. It is also unknown if TCR activation may play a direct role in promoting phagocytosis by macrophages expressing this receptor; while TCRs are required for antigen recognition by T cells and T cell activation, the effect of activating these receptors in macrophages is still unknown.

The expression of T-cell immunoglobulin and mucin-domain-containing molecule 3 (Tim-3) is reduced in monocytes from patients infected with *P. falciparum* and in splenic macrophages of *P. berghei*-infected mice [55]. Tim-3 is known to induce M2 macrophage polarization, hence its decreased expression may favour M1 macrophage activation to promote early parasite clearance; this remains to be proven. Although Tim-3 has been implicated in the clearance of apoptotic cells by binding to phosphatidylserine, it appears to inhibit the ability of CD11b^+^ mouse splenic macrophages to phagocytized *P. berghei*-infected cells [55].

**Fig. 2:**
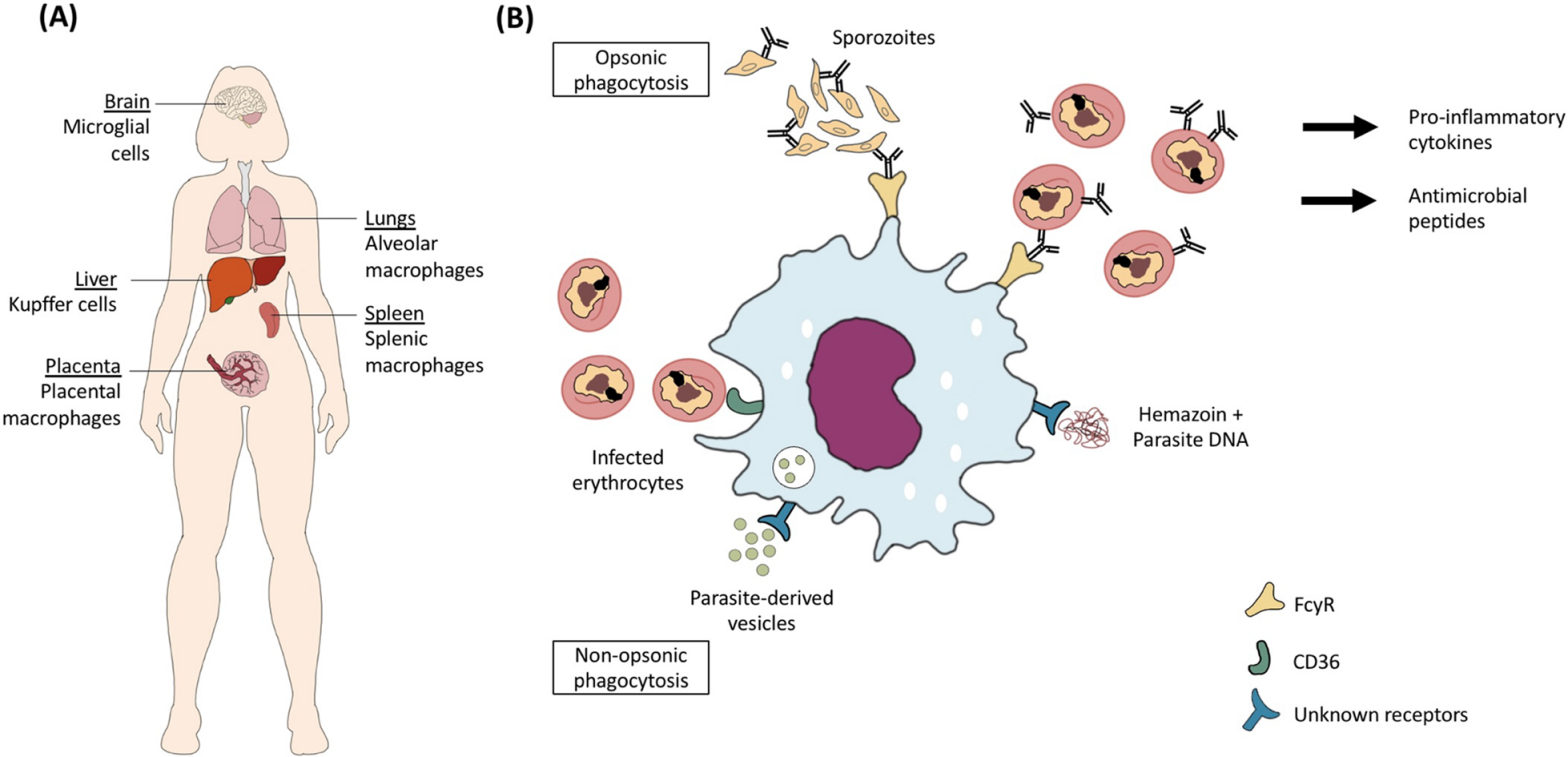
The role of macrophages in phagocytosis of *Plasmodium falciparum*. **A** There are different populations of macrophages that may interact with the parasites during an infection. Sporozoites encounter Kupffer cells in the liver during the initial stage of infection, and infected erythrocytes (IEs) circulated to the spleen for clearance will encounter splenic macrophages. In severe malaria, *P. falciparum* may encounter macrophages in other organs such as brain, placenta and lungs. **B** Macrophages can perform opsonic phagocytosis of IEs, which often results in the production of pro-inflammatory cytokines and antimicrobial peptides that play a role in inhibiting parasite growth [62, 63]. Similar to monocytes, they can directly uptake IEs using CD36, which is the non-opsonic phagocytosis pathway. Other phagocytic receptors involved in the uptake of haemozoin and parasite-derived vesicles are unknown

Macrophages reside in tissues, therefore they usually phagocytize mature-stage IEs (Fig. 2). The exception is for Kupffer cells, which serve as portal of entry for sporozoites to invade hepatocytes. These liver macrophages can phagocytize antibody-opsonized sporozoites, subsequently prevent the development of a blood-stage infection. Previous studies with different rodent models suggest that there are specific Kupffer cell populations that preferentially support either a protective or pathological role during sporozoites infection. One study reported that Kupffer cells that express CD68 support the entry of sporozoites to cause infection [56], while another showed that Kupffer cells that express triggering receptor expressed on myeloid cells 2 (TREM2) are able to reduce hepatocyte infection [57]. TREM2 is known to be an important phagocytic receptor that mediates the uptake of apoptotic cells, bacteria and lipids, as well as triggering pro-inflammatory pathways [58]. More studies are needed to elucidate its role during *Plasmodium* spp. infection.

Although there are interesting findings from rodent models, it is unknown whether these are fully translatable to human infections as there may be phenotypic and potentially functional differences between rodent and human macrophages. Within the spleen, red pulp macrophages, which are the most abundant macrophage population in the spleen, are involved in the clearance of aberrant and aged erythrocytes [59]. Rodent red pulp macrophages, characterized as F4/80^hi^Mac-1^low^ population, showed strong phagocytic activity for serum-opsonized zymosan (a structural component of yeast cell wall), suggesting that they are efficient at mediating opsonic phagocytosis [60]. On the other hand, human splenic red pulp macrophages are characterized as CD163^high^CD14^low^CD11b^−^ FcγRIIb^−^ population [61], and they also express other Fc receptors such as FcγRIIa and FcγRIIIa [61], but their ability to phagocytize IEs is not known. It is worth noting that for most tissue macrophages especially from humans, their immunological phenotypes and functions are not well understood. Hence, to enhance the understanding of the phagocytosis potential of these cells during *Plasmodium* spp. infection, it is useful to develop different humanized macrophage models or cell isolation protocols that allow distinct cell populations to be isolated wherever possible.

### Macrophage activation following phagocytosis

Similar to monocyte activation, one of the main downstream consequences following opsonic phagocytosis by macrophages is the production of pro-inflammatory mediators. Using monocyte-derived macrophages from malaria-naïve individuals, it was reported that phagocytosis of antibody-opsonized IEs resulted in the secretion of TNF and IL-1β, with the latter occurring due to the activation of inflammasome following FcγR engagement [62]. The phagocytosis of IEs by monocyte-derived macrophages also led to the up-regulation of β-defensin 130 (DEFB130), which is an antimicrobial peptide that can inhibit parasite growth in vitro [63]. In a rodent model, it was shown that haemozoin coupled to parasite DNA can be phagocytized by macrophages into phagolysosomes, with the haemozoin subsequently dissociating from the DNA and induce phagolysosomal destabilization [64]. This allows the nucleic acid to be released into the cytosol and bind to intracellular sensors such as TLR9 to initiate an inflammatory response, characterized by pro-inflammatory cytokine production [65].

In mouse models of experimental cerebral malaria (ECM), phagocytosis of parasite-derived vesicles by astrocytes and IEs by brain macrophages (microglia cells) led to increased production of inflammatory mediators such as CXCL-10 and high levels of CXCL-10 predicted mortality in ECM [66, 67]. To further corroborate the pathological role of CXCL-10 in malaria, elevated levels of this molecule were associated with increased risk of fatal cerebral malaria in children [68]. In a recent study, CD14^+^ monocytes were identified as the main producers of CXCL-10 when challenged with *P. falciparum*, but the sub-sets that were involved and the association between CXCL-10 levels and parasite burden in humans remain unknown [69]. In addition, using an in vitro model of human blood monocyte-derived microglia cells, the uptake of IE-derived extracellular vesicles was shown to cause down-regulation of IL-6 and IL-10 gene expression [70]. When mouse macrophages were challenged with these vesicles, they produced TNF and up-regulated their CD40 expression [71]. Extracellular vesicles appear to be important in mediating cell–cell communication and immune cell activation, hence further research is needed to understand the mechanisms and receptors involved in the uptake of these vesicles.

### Neutrophils: the circulating and most abundant phagocytes

#### Neutrophil sub-sets

Neutrophils are the most abundant leukocytes in circulation and they express high levels of Fcγ receptors. Of the three FcγRs, they express highest levels of FcγRIIIB [72]. Neutrophils have been shown to phagocytize antibody-opsonized merozoites, haemozoin and IEs, however, the association between neutrophil phagocytosis and protection against malaria is not well understood. Similar to monocytes and macrophages, heterogeneity within the neutrophil population has also been recognized [73]. They can switch their phenotype in response to stimuli such as GM-CSF and can be primed to enhance certain immune functions such as phagocytosis [74]. Neutrophils exist in different maturation states within peripheral blood, including immature (with band-shaped nuclei), mature (with segmented nuclei) and aged (with hypersegmented nuclei) neutrophils. Immature neutrophils are usually present in the bone marrow, but they may be detected in the peripheral blood during state of infection and inflammation, including malaria [75, 76]. Mature neutrophils are terminally differentiated in the bone marrow before being released into the circulation, while aged neutrophils are eventually cleared by macrophages in the liver and spleen [77]. Although these neutrophil populations appear different in their phenotypes, it is unknown if they play distinct or overlapping roles in malaria.

### Neutrophil sub-sets and their phagocytosis function

Circulating neutrophils appear to be a heterogeneous population that exhibit either high and/or low phagocytic potentials; however, it remains challenging to characterize and differentiate these cells in humans [78]. A specific sub-population of mouse TCRβ^+^ neutrophils were reported to be highly efficient in in vitro phagocytosis of *P. berghei*-IEs, with fourfold greater efficiency compared to TCRβ^−^ neutrophils [79]. In the human population, it was reported that approximately 5 to 8% of human neutrophils express this receptor [80]. However, it is unknown if the highly phagocytic neutrophil sub-set is associated with protection from clinical and severe malaria.

Neutrophils have been further classified into high- and low-density neutrophils, with the latter being found in the peripheral blood of cancer patients and those with HIV infection [83]. While low-density neutrophils may comprise immature neutrophils and activated mature neutrophils, these sub-populations remain poorly defined, both phenotypically and functionally [83]. Increased proportions of low-density neutrophils/granulocytes have been reported in patients with *P. vivax* infection, where the authors suggested that these cells were comprised of activated degranulated neutrophils [76]. Although the low-density neutrophils exhibited decreased ability in phagocytizing bacterial cells [81], they were reported to have high ability in the uptake of *P. vivax*-infected reticulocytes [76]. In another study, neutrophils isolated from *P. vivax*-infected patients also showed higher phagocytosis ability compared to neutrophils isolated from healthy donors [82]. The authors used opsonized zymosan as phagocytic targets, hence *Plasmodium* spp. infection may have up-regulated FcγR expression on neutrophils to enhance opsonic phagocytosis.

### Neutrophil activation following phagocytosis

Neutrophils can generate reactive oxygen species (ROS) when activated, and these highly toxic molecules can kill parasites by causing oxidative damage. ROS levels have been shown to be associated with protection against fatal cerebral malaria and clinical malaria episodes [83, 84]. Interestingly, ROS production was induced when neutrophils were stimulated with opsonized merozoites and parasite digestive vacuoles, but was undetectable when opsonized IEs were used as targets [85]. This suggests that the induction of ROS may depend on specific parasite antigens, and previous studies demonstrated high levels of antibodies targeting MSP-1, MSP-4 and MSP-5 to be correlated with ROS production [83, 86].

Uptake of unopsonized IEs by neutrophils in vitro resulted in increased pro-inflammatory responses, characterized by the up-regulation of mitogen-activated protein kinase, interleukin and interferon-gamma signalling pathways [87]. Interestingly, this is in contrast to non-opsonic phagocytosis of parasites by monocytes/macrophages, which does not induce production of pro-inflammatory mediators. Future studies investigating the underlying reasons for this difference are needed to better understand the immunomodulatory effect of parasites on phagocytes.

Another function of neutrophils in defence against *Plasmodium* spp. is neutrophil extracellular traps (NETs) formation via a type of programmed cell death known as NETosis. This process results in the release of neutrophil intracellular content, including decondensed chromatin coupled with granular proteins and histones that can trap pathogens. Monosodium urate crystals and haem are two products released into the blood circulation following IEs rupture and both can cause the release of NETs [88, 89]. Phagocytosis of monosodium urate crystals involves actin cytoskeleton remodelling and this process is required to promote NETosis [90]. However, it is currently unknown if haem similarly needs to be phagocytized to trigger NETosis, or the surface receptor–ligand interaction is sufficient to induce the release of NETs. Although NETs are useful in limiting the proliferation and dissemination of pathogen, they were reported to induce up-regulation of endothelial cytoadhesion receptors, leading to increased parasite sequestration in mice malaria [89]. This can potentially cause vaso-occlusion and increased disease severity observed in severe malaria cases [89].

## Parasite haemozoin can stimulate phagocytosis

When IEs rupture, haemozoin crystals are released and taken up by phagocytes. The phagocytosis of haemozoin by neutrophils was recently reported to be dependent on the presence of platelets and can be inhibited by host fibrinogen [91]. Neutrophils that have phagocytized haemozoin have been associated with severe and poor clinical outcomes in *P. falciparum* [8, 92] and *P. vivax* infections [76]. Similarly, monocytes that have phagocytized haemozoin have been associated with severe malaria in children and low birth weight in placental malaria [7, 93]. Post-mortem analysis of *P. falciparum*-infected organs often show the presence of haemozoin-laden macrophages, indicating that they are well capable of phagocytosis. However, haemozoin cannot be degraded in the phagocytes and often results in the impairment of phagocyte function, where they do not repeat the phagocytic cycle after ingesting the haemozoin, thus reducing phagocytosis capacity [9].

## Specific interaction of parasites with host phagocytes

*Plasmodium* spp. can also manipulate its interaction with host phagocytic receptors and the downstream signalling pathways to escape phagocytosis. For example, in *Plasmodium yoelii* mouse malaria model, the parasites preferentially infect erythrocytes that express high levels of CD47 [94], allowing them to escape phagocytosis by red pulp macrophages in the spleen. CD47 is a self-marker that inhibits phagocytosis by interacting with macrophage signal-regulatory protein alpha, hence decreased CD47 can promote phagocytic clearance. *Plasmodium falciparum*- and *P. vivax*-IEs were reported to express higher levels of CD47 compared to uninfected erythrocytes, however, the mechanism of this higher expression is unknown [95]. In addition, the parasites can also evade phagocytosis by acquiring complement regulatory proteins, whose function is to protect host cells against complement-mediated damage. Merozoites and IEs can actively recruit and use host regulatory proteins such as Factor H and Factor H-like protein to inactivate C3b on the surface of IEs, hence prevent complement-mediated phagocytic clearance of the parasites [96, 97].

Another example is the evasion of phagocytosis through antigenic switching by placental-binding IEs. Parasites found in the placenta commonly attach to chondroitin sulfate A and are unable to bind CD36, a macrophage phagocytic receptor, hence they accumulate in the placenta and cause pathologies [98]. This is particularly true in primigravids, who have yet to develop protective antibody immunity. During infection, the expression of complement receptor 1 (CR1) by monocytes/macrophages is down-regulated [99], leading to high levels of immune complexes such as those observed in patients with severe anaemia and cerebral malaria. CR1 binds to immune complexes and causes them to be phagocytized, which otherwise can cause pathologies due to activation of immune responses. Interestingly, IEs also preferentially bind to CR1 expressed by uninfected erythrocytes to form rosettes, which may potentially shield them from phagocyte recognition. A recent study reported that IEs can stimulate monocytes to produce insulin growth factor binding protein 7, which promotes rosette formation and subsequently inhibits monocyte phagocytosis [100]. Non-rosetting *P. vivax* IEs were also shown to be more likely to be phagocytized by THP-1 macrophages [101]. Lastly, parasites may directly induce apoptosis of phagocytes, as shown in Kupffer cells during rodent malaria [102].

## Host inflammatory mediators

In response to malaria parasites, host immune cells can produce a variety of cytokines that can direct the activation or priming status of phagocytes, hence influence their phagocytic ability. For example, IFN-γ is known to enhance opsonic phagocytosis of IEs by increasing the expression of FcγRI [103], which is beneficial for parasite clearance. Early and high production of IFN-γ has been associated with protection against symptomatic malaria [104]. In vitro treatment of human neutrophils with various pro-inflammatory cytokines (IFN-γ, IL-1β, TNF and GM-CSF) was reported to increase phagocytosis of opsonized merozoites [15], while priming with IFN-γ enables them to efficiently phagocytize unopsonized IEs [87]. Furthermore, in rodent models, treatment with IFN-γ or TNF was reported to enhance the phagocytosis of unopsonized merozoites by peritoneal macrophages, whereas IL-10 treatment was reported to significantly reduce the phagocytosis potential of these macrophages [105]. It remains difficult to determine if the cytokines are contributing to either disease protection or pathology by modulating phagocytosis or through other mechanisms, given that each cytokine may exhibit pleiotropic effects.

## Underlying red cell disorders and blood group

Red cell disorders, such as thalassemia, glucose-6-phosphate dehydrogenase (G6PD) deficiency and sickle cell trait have been associated with protection against severe malaria [106–108]. One of the proposed mechanisms of protection is the increased and early phagocytic clearance of IEs in these individuals. Increased deposition of autologous IgG and complement C3c fragments, as well as membrane-bound hemichromes and aggregate band 3, was found in ring-stage IEs from individuals with sickle trait and beta-thalassemia trait; these promote phagocytosis of ring-stage parasites by human monocytes [109]. Similarly, ring-stage IEs that were deficient in G6PD were also more efficiently phagocytized compared to normal infected erythrocytes due to higher levels of autologous IgG and complement C3 fragments [110]. A recent study reported that sickle cells have a higher expression of terminal mannose, which can be recognized by mannose receptors on macrophages and promote phagocytosis [111]. Interestingly, it was also reported that *P. falciparum*-infected O erythrocytes were taken up more efficiently by human macrophages in vitro and mouse monocytes in vivo compared to infected A and B erythrocytes [112], suggesting that this could be one of the mechanisms by which individuals with blood type O are protected against severe malaria.

## Human immunodeficiency virus (HIV) co-infection

HIV infection is prevalent in many malaria-endemic regions, leading to cases of co-infection. Studies investigating immune responses in these individuals suggest that phagocytosis of IEs may be compromised via several mechanisms. Reduced levels of opsonizing antibody to both placental-binding and non-placental-binding IEs have been associated with HIV infection [113, 114]. In addition, HIV-infected macrophages have reduced capacity to phagocytize IEs and secrete lower quantities of pro-inflammatory cytokines [115]. HIV infection is also associated with reduced expression of FcγRs on macrophages [116], potentially reducing opsonic phagocytosis of parasites. These studies imply that co-infection with HIV can cause impaired parasite clearance, subsequently leading to adverse malaria outcomes.

## Anti-malarials and antibiotics: beyond their anti-microbial functions

Several in vitro studies showed that anti-malarial drugs and antibiotics administered to *Plasmodium* spp.-infected patients can modulate host immune cell phagocytosis function. An older study reported that IEs treated with anti-malarials, such as chloroquine, quinine, amodiaquine, and mefloquine, can down-regulate IgG opsonization of these cells and prevent phagocytosis [117]. A recent study reported that artemesinin, quinine, primaquine, and pyrimethamine can increase the phagocytosis of synthetic haemozoin by monocytes, while amodiaquine, chloroquine and doxycycline had opposing effect [118]. The development of resistance by the malaria parasites against existing anti-malarials has led to the development of new drug formulations, including use of antibiotics that may have immunomodulatory effects. For example, studies have trialled the use of azithromycin as a potential anti-malarial drug and this antibiotic has been shown to promote phagocytosis of apoptotic cells by alveolar macrophages [119]. Furthermore, many of these drugs have anti-inflammatory properties and more studies need to be done to understand how they may affect phagocytosis of *Plasmodium*.

## Modulation of phagocytosis function

Knowledge of the molecular interactions between phagocytes and parasites is useful in the design of therapeutics that can modulate their phagocytosis function. To date, three promising immunomodulatory compounds, namely curcumin, rosiglitazone and Vitamin A, have been shown to increase phagocytosis receptor expression and phagocytosis of parasites [120–122]. Inhibitory molecules that target specific immune pathways may also be useful in altering the phagocytic function of phagocytes. For example, PD98059, a MEK (mitogen-activated protein kinase kinase) 1/2 inhibitor, was reported to enhance the clearance of parasites by splenic and liver macrophages and neutrophils by increasing phagocytic receptor expression such as CD36, MARCO and complement receptor 1/2 [123]. Furthermore, the phagocytic process can be regulated by Rab14, a GTPase whose expression was shown to be up-regulated in mouse macrophages after *P. berghei* infection [124]. Rab14, which localizes to early endosomes, can mediate phagosome maturation and internalization of pathogens in murine bone marrow-derived macrophages, which in turn decreases CD36 expression and suppresses phagocytosis [124, 125]. Future studies are needed to investigate whether human malaria infection affects Rab14 expression and if this has any effect on parasite clearance. If phagocytosis is suppressed, then Rab14 inhibitor proteins can potentially improve phagocytosis and promote parasite clearance.

Given the pathological role of M1 and M2 macrophages in various inflammatory diseases, drugs and nanoparticles specifically targeting the function of these immune cells have been designed as treatment options [126]. The therapy aims to alter the polarization status, recruitment and function of macrophages, including cytokine production and phagocytosis. Many natural compounds also have the ability to promote or alter the polarization status of macrophages but so far, none of these has been tested in the malarial setting [127]. Host defence peptides such as cathelicidin are also potential immunomodulatory molecules. They can be administered as synthetic peptides or by inducing their production through the consumption of Vitamin D or butyrate [128, 129]. One of these peptides, LL-37, has been reported to up-regulate phagocytosis levels by increasing the expression of Fcγ receptors I and II, in addition to acting as opsonins for gram positive and negative bacteria [130, 131]. However, the role of these antimicrobial peptides in regulating phagocytosis during *Plasmodium*-infection has yet to be investigated.

## Concluding remarks and future perspectives

The understanding of factors that contribute to the success of phagocytosis in controlling the malaria parasite is still rather limited; herein, priorities for future research are listed in Table 2. With major advancement in cell-targeted immunomodulatory therapy, enhanced knowledge of phagocyte functions during malaria is likely to be beneficial in the design of new therapeutics. In particular, understanding the role of different sub-populations of phagocytes, whether they contribute to protection or pathology, is critical. For future studies aimed at assessing the role of phagocytosis during *Plasmodium* infection, it is important to note that besides the availability of opsonins, the presence of the protective phagocyte sub-populations, if they exist, is also of paramount importance.

**Table 2:** Priorities for future research in understanding phagocytosis during *Plasmodium* infection

**Phagocytes and clinical protection**
Does high phagocytosis potential of specific immune cells or their subpopulations correlate with protection and disease progression?
What parameters can be used to define ‘effective phagocytosis’ level (level that is sufficient to confer protection against clinical disease)?
Can host genetic variations lead to different expression levels of phagocytic receptors, and will these impact phagocytic potentials of phagocytes, hence leading to different disease susceptibility?
How are the expression of opsonic and non-opsonic phagocytosis receptors regulated during malaria infection and do these expressions correlate with protection/pathology?
**Molecular mechanisms of phagocytosis**
Are there specific phagocyte population or subpopulations that are responsible for clearing different stages of the parasites in their asexual life cycle?
Which parasite ligands specifically interact with the phagocytes, leading to immunomodulation of phagocytosis?
What are the other phagocytic receptors involved in non-opsonic phagocytosis of *Plasmodium* and what are the downstream consequences of this interaction?
What are the roles of host defence peptides in phagocytosis during malaria infection?
**Immune activation following phagocytosis**
Are there adverse consequences that may result from increased phagocytosis, such as activation of inflammation that results in endothelial-lining damage and host pathology?
**Modulation of phagocytosis**
How do chronic or co-infections, such as HIV, hepatitis or helminth infections, affect the phagocytic function of phagocytes in infected hosts?
Can specific compounds or therapeutics with the potential to increase the rate phagocytosis improve treatment outcomes and/or reduce clinical manifestations of malaria?
Will immunoregulatory therapeutics aimed at dampening immune responses during severe malaria decrease phagocytic capability and thus, reduce the efficiency of parasite clearance?
What are the specific immune evasion strategies employed by *Plasmodium* parasites to avoid phagocytosis by phagocytes?

## Data Availability

Not applicable.

## Abbreviations

CR1: Complement receptor 1
CSP: Circumsporozoite protein
DEFB130: β-Defensin 130
ECM: Experimental cerebral malaria
G6PD: Glucose-6-phosphate dehydrogenase
GM-CSF: Granulocyte/macrophage colony stimulating factor
HIV: Human immunodeficiency virus
IEs: *Plasmodium*-infected erythrocytes
IFN: Interferon
IgG: Immunoglobulin
MSP: Merozoite surface protein
MSPDBL: MSP-Duffy binding-like
NETs: Neutrophil extracellular traps
PfEMP1: *Plasmodium falciparum* erythrocyte membrane protein 1
ROS: Reactive oxygen species
STING: Stimulator of interferon genes
TCR: T cell receptor
TIM-3: T-cell immunoglobulin and mucin-domain-containing molecule-3
TLR: Toll like receptor
TNF: Tumour necrosis factor
TREM2: Triggering receptor expressed on myeloid cells2
